# Differential Mechanisms of Ang (1-7)-Mediated Vasodepressor Effect in Adult and Aged Candesartan-Treated Rats

**DOI:** 10.1155/2012/192567

**Published:** 2011-11-30

**Authors:** S. Bosnyak, R. E. Widdop, K. M. Denton, E. S. Jones

**Affiliations:** ^1^Departments of Pharmacology, Monash University, Clayton, VIC 3800, Australia; ^2^Departments of Physiology, Monash University, Clayton, VIC 3800, Australia

## Abstract

Angiotensin (1-7) (Ang (1-7)) causes vasodilator effects in Wistar-Kyoto (WKY) rats and spontaneously hypertensive rats (SHRs) via angiotensin type 2 receptors (AT_2_R). However, the role of vascular AT_2_R in aging is not known. Therefore, we examined the effect of aging on Ang (1-7)-mediated vasodepressor effects and vascular angiotensin receptor localization in aging. Blood pressure was measured in conscious adult (*~*17 weeks) and aged (*~*19 months) normotensive rats that received drug combinations in a randomised fashion over a 4-day protocol: (i) Ang (1-7) alone, (ii) AT_1_R antagonist, candesartan, alone, (iii) Ang (1-7) and candesartan, or (iv) Ang-(1-7), candesartan, and the AT_2_R antagonist, PD123319. In a separate group of animals, the specific *Mas*R antagonist, A779, was administered in place of PD123319. Receptor localisation was also assessed in aortic sections from adult and aged WKY rats by immunofluorescence. Ang (1-7) reduced blood pressure (*~*15 mmHg) in adult normotensive rats although this effect was dependant on the background dose of candesartan. This depressor effect was reversed by AT_2_R blockade. In aged rats, the depressor effect of Ang (1-7) was evident but was now inhibited by either AT_2_R blockade or *Mas*R blockade. At the same time, AT_2_R, *Mas*R, and ACE2 immunoreactivity was markedly elevated in aortic sections from aged animals. These results indicate that the Ang (1-7)-mediated depressor effect was preserved in aged animals. Whereas Ang (1-7) effects were mediated exclusively via stimulation of AT_2_R in adult WKY, with aging the vasodepressor effect of Ang (1-7) involved both AT_2_R and *Mas*R.

## 1. Introduction 

It is well known that Angiotensin II (Ang II) mediates its physiological functions via two main receptor subtypes, the type 1 (AT_1_R) and type 2 (AT_2_R) angiotensin receptors where it has similar affinity for both the AT_1_R and AT_2_R. However, there is now increasing evidence suggesting that angiotensin peptides other than Ang II can evoke cardiovascular effects that oppose the effects mediated by the AT_1_R via a number of non-AT_1_R mechanisms. In fact, heptapeptide Angiotensin (1-7), (Ang (1-7)), a biologically active metabolite of angiotensin I (Ang I) and Ang II [1, 2] has been shown to possess biological activity in its own right [3]. Interest in Ang (1-7) has surged since the discovery of angiotensin converting enzyme type 2 (ACE2) and recognition that Ang (1-7) can be produced directly from Ang II via ACE2 [1, 2]. Although, Ang (1-7) differs to Ang II by only one amino acid, Ang (1-7)-mediated effects are markedly different to those of Ang II, and it has been suggested that Ang (1-7) may in fact play a counterregulatory role to Ang II [4], mediating a range of effects such as vasodilatation, inhibition of vascular smooth muscle proliferation, and fluid and electrolyte homeostasis [5]. The cardiovascular effects of Ang (1-7) are often reported to be inhibited by the D-Ala^7^ Ang (1-7) analogue, known as A779 [6]. Recently, Ang (1-7) was identified as an endogenous ligand for the Ang (1-7)/*Mas*R (*Mas*R), since Ang (1-7)-mediated vasorelaxation was impaired in *Mas*R^−/−^ mice [7]. However, under some circumstances, Ang (1-7) can mediate its effects via AT_2_R [8–10]. In fact, we have shown that Ang (1-7)-mediated vasodepressor effect was via an AT_2_R sensitive pathway [11]. In that study, Ang (1-7) acutely lowered blood pressure in spontaneously hypertensive rats (SHRs) and Wistar-Kyoto (WKY) adult rats during concomitant AT_1_R blockade, [11] in a similar manner to that seen with AT_2_R agonist, CGP42114 [12, 13], and more recently with selective nonpeptide AT_2_R agonist, Compound 21 [14]. Furthermore, the AT_2_R antagonist, PD123319, but not the *Mas*R antagonist, A779, blocked this vasodepressor effect of Ang (1-7) [11].

While it is well recognized that the renin-angiotensin system (RAS) has a critical role in the cardiovascular system; its role in the aging process is still under investigation. During aging, circulating levels of Ang II are downregulated while local production of Ang II is increased in the aorta and other vessels [15] suggesting an essential role of local RAS in the vasculature during aging. However, there is little functional evidence about angiotensin receptors and their role during aging. In this context, we have shown that AT_2_R expression was increased in both endothelial and vascular smooth muscle of aortae obtained from aged WKY rats [16]. 

Given that there was an increased vascular AT_2_R expression in aging [16], the current study was designed to test our hypothesis that AT_2_R-mediated depressor function was preserved with aging. In the present study, we have used Ang (1-7) as an endogenous ligand for the AT_2_R, as we have previously reported in adult rats [11]. In preliminary experiments, we have determined that vascular expression of both AT_2_R and *Mas*R/ACE2 axis was upregulated with aging. Therefore, this strategy of using Ang (1-7) will also determine whether or not there was a role for *Mas*R to evoke vasodepressor effects with aging.

## 2. Methods

### 2.1. Animals

All animal care and experimental procedures were approved by the Monash University Animal Ethics Committee and performed according to the guidelines of the National Health and Medical Research Council of Australia for animal experimentation. 

16- to 18-week-old WKY male rats (300 to 350 g) and 20-month-old WKY male rats (450–500 g) were obtained from the Animal Resource Centre (Perth, Wash, USA) and were used to represent adult and aged normotensive rats, respectively. Animals were maintained on a 12-hour day/night cycle with standard laboratory rat chow and water available *ad libitum*.

### 2.2. *In Vivo* Procedures

Rats were anesthetised (ketamine and xylazine; 75 mg/kg and 10 mg/kg, i.p, resp.; supplemented as required). Two catheters were inserted into the right jugular for intravenous drug administration. A catheter was implemented into the right carotid artery for direct blood pressure measurement as described previously [11–14]. Rats were housed in individual cages and allowed free access to food and water while maintained on 12-hour day/night cycle. The arterial catheter was infused overnight with heparinised saline using an infusion pump.

24 hours after the surgery, the arterial catheter was attached to a pressure transducer (Gould Inc), connected to a MacLab-8 data acquisition system (ADInstruments, Sydney), interfaced to a Macintosh computer. Mean arterial pressure (MAP) and heart rate (HR) were computed from the phasic blood pressure signal.

### 2.3. Experimental Protocol

Rats received drug combinations in a randomised fashion over a 4-day protocol, as we have performed previously [11, 12, 14]. Doses for candesartan and PD123319 were chosen on the basis of previous studies [11, 12, 14]. Six groups of rats underwent experimental protocols during which basal MAP and HR were recorded. Adult and aged WKY rats (Groups 1 and 2, resp.) were randomized to receive the following treatments on different days: (1) candesartan (0.01 mg/kg), (2) Ang (1-7) infusion (15 pmol/kg per minute for 4 hours), (3) Ang (1-7) infusion together with candesartan, and (4) a 4-hour infusion (0.1 mL/kg per hour IV) of saline (0.9% NaCl) to confirm a lack of effect on MAP. Animals in Group 3 (adult WKY rats) and Group 4 (aged WKY rats) were randomized to receive the following treatments: (1) candesartan at a 10-fold higher dose (0.1 mg/kg), (2) Ang (1-7) infusion (15 pmol/kg per minute for 4 hours), (3) Ang (1-7) infusion together with candesartan, and (4) Ang (1-7) infusion in the presence of candesartan and PD123319 infusion (50 *μ*g/kg per minute for 2 hours). In analogous experiments in additional adult and aged WKY rats (Groups 5 and 6), the putative Ang (1-7) antagonist, A779 (15 pmol/kg per minute), was used instead of PD123319. Doses of Ang (1-7) and A779 are based on our previous study [11].

### 2.4. Localization of ACE2, AT_**1**_, AT_**2**_, and *Mas* Receptors

Localization of ACE2, AT_1_, AT_2_, and *Mas* receptosrs using immunofluorescence was performed using thoracic aortic sections taken from naïve aged and adult rats to determine changes in expression levels between the two age groups. Male adult and aged WKY rats were killed by isoflurane inhalation followed by decapitation, and the thoracic aorta was removed in order to dissect 3–5 mm long sections. Immunofluorescence was performed using 10 *μ*m thick section of thoracic aorta cut on Cryostat. Aortic sections were incubated overnight at 4°C with 1/500 dilution of polyclonal rabbit antibodies raised against AT_1_R, AT_2_R, *Mas*R, and ACE2. Following overnight incubation, sections were incubated for 2.5 hour with a goat anti-rabbit secondary antibody conjugated with Alexa 568 flurophore. Rabbit IgG antibody was used as negative control. Sections were mounted with antifade medium (VectorShield) and cover slipped. Sections were imaged using Olympus Fluoview 500 confocal microscope equipped with a krypton/argon laser. Fluorescence intensity was quantified using analysis professional software (Soft Imaging System, Singapore) with identical measurement settings.

### 2.5. Statistical Analysis

All data are presented as mean responses ± standard error of the mean (SEM). Differences in MAP between treatments were analysed using a 2-way ANOVA repeated measure analysis of variance. Differences in fluorescence intensity were analysed using 1-way ANOVA with Bonferroni corrections where appropriate. Statistical analysis was performed using GraphPad Prism (Version 5.0c). *P* values <0.05 were deemed statistically significant.

### 2.6. Materials

PD123319 and candesartan were kind gifts from Pfizer and AstraZeneca, respectively. All other chemicals were purchased from commercial sources: ketamine (Troy Laboratories, Australia), xylazine (Troy Laboratories), isoflurane (Baxter, USA), Ang (1-7) (Ausep, Australia), A779 (Auspep, Australia), rabbit polyclonal antibodies raised against AT_1_R, AT_2_R, and ACE2 (Santa Cruz Biotechnology Inc., Catalogue no. SC1173, SC9040, and SC2099), rabbit polyclonal antibody raised against *Mas*R (Novus Biologicals, USA, Catalogue no. NLS1531), secondary goat anti-rabbit Alexa 568 antibody (Invitrogen, USA, Catalogue no. A-21069).

## 3. Results

### 3.1. *In Vivo* Effect of Ang (1-7) in Conscious Normotensive Rats

Basal MAPs of WKY rats over the 4 experimental days for each group are listed in Table 1. There was no significant difference between resting MAPs over the experimental period for any of the treatment groups, suggesting that none of the acute treatments had long-lasting effects and, therefore, did not influence baseline MAP on subsequent days.

**Figure 1:**
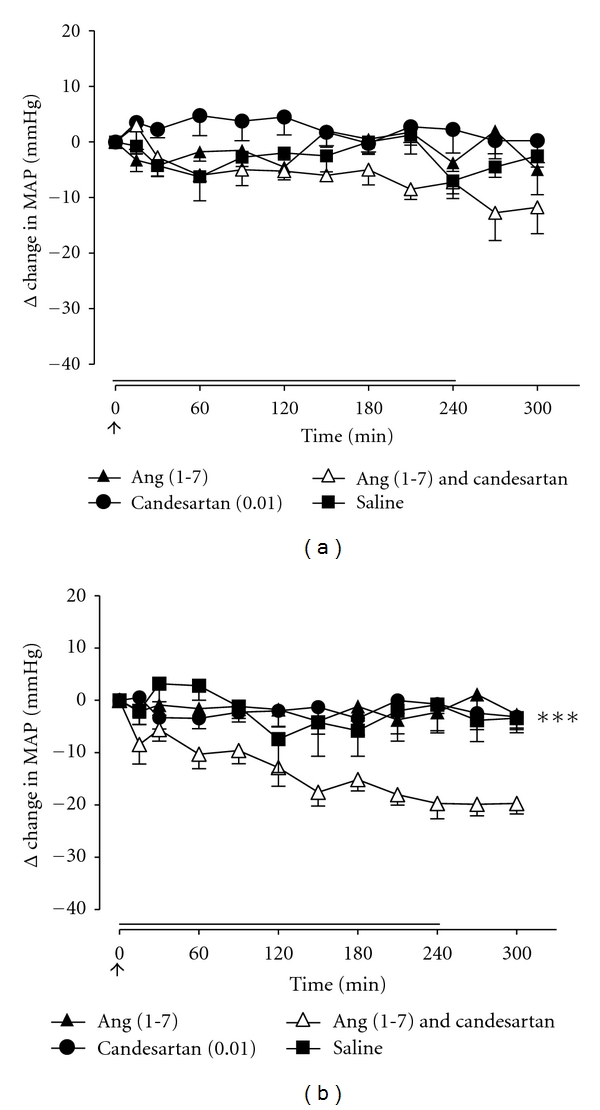
Effect of the AT_1_R Ang (1-7) (15 pmol/kg/min; 4-hour infusion depicted by full line), AT_1_R antagonist, candesartan (0.01 mg/kg bolus IV; depicted by an arrow), saline (0.1 mL/kg 0.9% NaCl IV for 4 hours), and Ang (1-7) + candesartan on MAP in (a) adult WKY rats (*n* = 4) and (b) aged WKY rats (*n* = 7). Values represent mean ± SEM. ***P < 0.001, for treatment effect of Ang (1-7) + candesartan versus all other treatments (2-way RM ANOVA).

**Figure 2:**
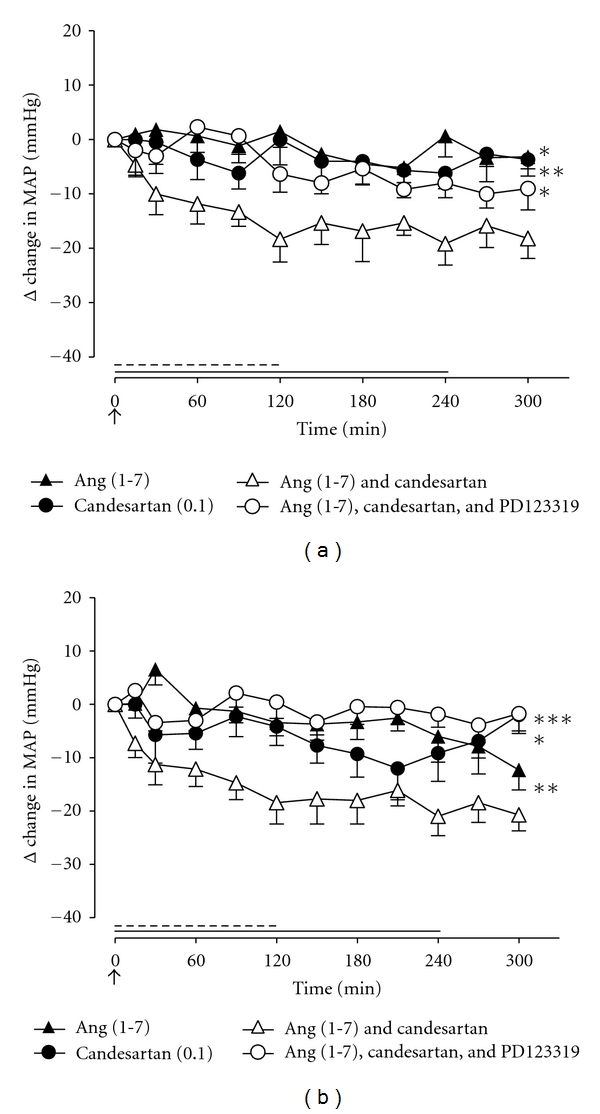
Effect of Ang (1-7) (15 pmol/kg/min; 4-hour infusion depicted by full line), AT_1_R antagonist, candesartan (0.1 mg/kg bolus IV; depicted by an arrow), Ang (1-7) + candesartan, and Ang (1-7) + candesartan + AT_2_R antagonist, PD123319 (50 *μ*g/kg/min for 2 hours; depicted by dashed line), on MAP in (a) adult WKY rats (*n* = 6) and (b) aged WKY rats (*n* = 7). Values represent mean ± SEM. *P < 0.05; **P < 0.01; ***P < 0.001, for treatment effect of Ang (1-7) + candesartan versus all other treatments as indicated (2-way RM ANOVA).

In groups 1 and 2, infusion of saline had no significant effect on MAP (Figure 1). Therefore, this treatment was not performed in subsequent groups in order to include additional treatment arms. In all groups, infusion of Ang (1-7) (15 pmol/kg/min) or candesartan (0.01 or 0.1 mg/kg IV) had no significant effect on MAP. Coinfusion of Ang (1-7) and candesartan (0.01 mg/kg IV) had no effect on MAP in adult WKY rats (Figure 1(a)) whereas, in aged WKY rats, combined administration of Ang (1-7) and candesartan (0.01 mg/kg IV) significantly decreased MAP (*P* < 0.001) (Figure 1(b)). When Ang (1-7) was combined with a 10-fold higher dose of candesartan (0.1 mg/kg IV), there were significant reductions in MAP in both adult and aged WKY male rats compared with Ang (1-7) alone or candesartan alone (*P* < 0.01). Moreover, this depressor effect of Ang (1-7) was abolished by the addition of the AT_2_R antagonist, PD123319 (50 *μ*g/kg/min), (Figures 2(a) and 2(b)). 

**Figure 3:**
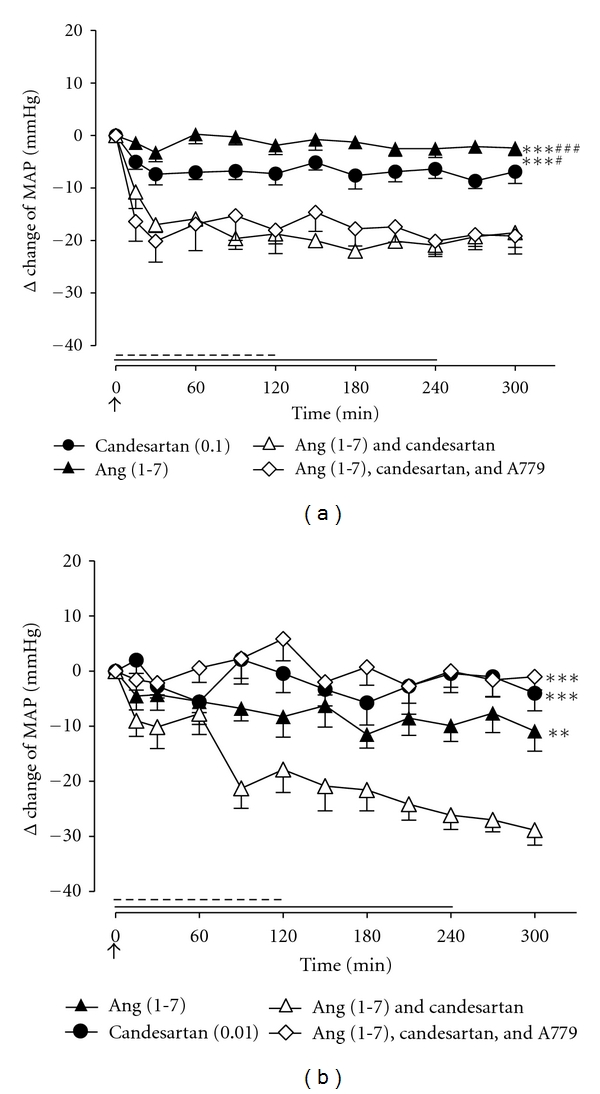
Effect of Ang (1-7) (15 pmol/kg/min; 4-hour infusion depicted by full line), AT_1_R antagonist, and candesartan in (a) adult WKY rats (0.1 mg/kg bolus IV; depicted by an arrow) and (b) aged WKY rats (0.01 mg/kg bolus IV; depicted by an arrow), together with Ang (1-7) + candesartan and Ang (1-7) + candesartan + *Mas*R antagonist A779 (15 pmol/kg/min for 2 hours; depicted by dashed line), on MAP (*n* = 8 for both groups). Values represent mean ± SEM. (a) ***P < 0.001, for treatment effect of Ang (1-7) + candesartan versus Ang (1-7) or candesartan alone (2-way RM ANOVA), ^†^<0.05; ^†††^<0.001, for treatment effect of Ang (1-7) + candesartan +A779 versus candesartan or Ang (1-7) alone (2-way RM ANOVA). (b) **P < 0.01; ***<0.001 for treatment effect of Ang (1-7) + candesartan versus Ang (1-7), candesartan, or Ang (1-7) + candesartan + A779 (2-way RM ANOVA).

In separate groups of animals, we examined the ability of the *Mas*R antagonist A779 to modify the Ang (1-7)-mediated depressor effect. Coinfusion of the Ang (1-7) antagonist A779 with the Ang (1-7)/candesartan combination in adult WKY male rats did not affect Ang (1-7)-mediated depressor response (Figure 3(a)). By contrast, the Ang (1-7)-evoked depressor response, during AT_1_R blockade, in aged WKY rats was in fact abolished by the addition of A779 (Figure 3(b)).

### 3.2. Localization of ACE2, AT_**1**_, AT_**2**_, and *Mas* Receptors

**Figure 4:**
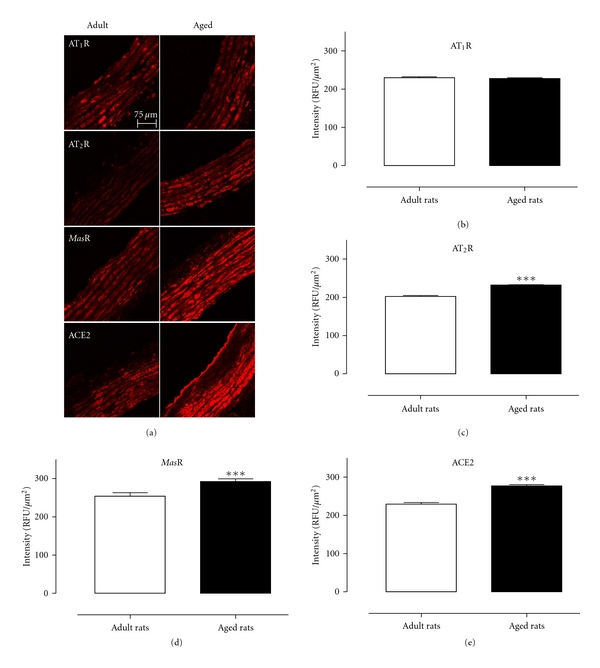
(a) Representative immunolocalisation images of AT_1_R, AT_2_R, *Mas*R, and ACE2 in adult WKY rats and aged WKY rats. Mean data for aortic expression of the (b) AT_1_R, (c) AT_2_R, (d) *Mas*R, and (e) ACE2 expressed as relative fluorescent units in adult (*n* = 5) and aged (*n* = 4) WKY rats. ***P < 0.001 versus adult WKY rats.

**Figure 5:**
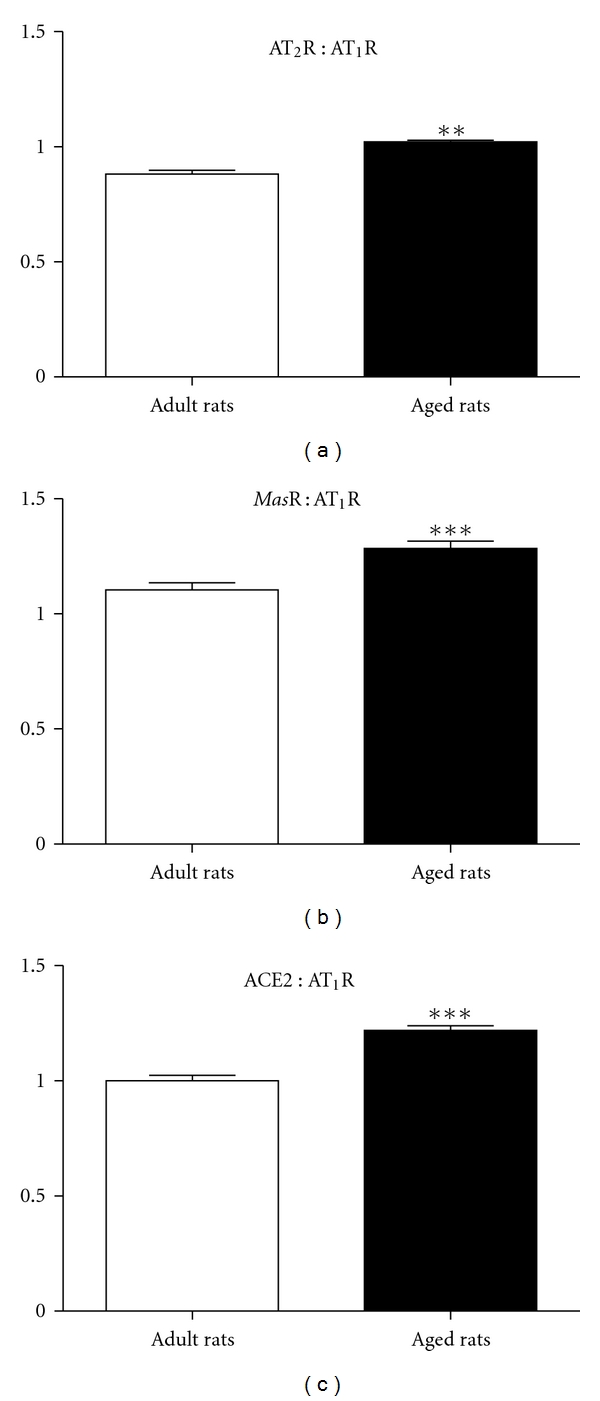
Ratios of (a) AT_2_R : AT_1_R, (b) *Mas*R : AT_1_R, and (c) ACE2 : AT_1_R in adult and aged WKY rats. **P < 0.01; ***P < 0.001 versus adult WKY rats.

Expression levels of ACE as well as angiotensin levels were determined using thoracic sections taken from naïve adult WKY rats (*n* = 5) and aged WKY rats (*n* = 4). ACE2, AT_1_R, AT_2_R, and *Mas*R were all localised throughout the entire aortic sections (Figure 4(a)). Expression levels of the AT_1_R were not changed between adult and aged WKY male rats, whereas ACE2, AT_2_R, and *Mas*R expression levels were all significantly upregulated in aged WKY rats compared to adult WKY rats (Figures 4(b)–4(e)). Therefore, when expressed relative to AT_1_R levels, each of the vasodilator non-AT_1_R components of the RAS was significantly increased in aged WKY rats compared to adult WKY rats (Figure 5).

## 4. Discussion

The main findings of the current study demonstrate for the first time that the depressor effect evoked by Ang (1-7) is preserved in aged normotensive candesartan-treated animals and was sensitive to both AT_2_R and *Mas*R blockade which contrasts with the involvement of only AT_2_R in the effects of Ang (1-7) in adult candesartan-treated rats. Moreover, these findings were consistent with increased AT_2_R, *Mas*R, and ACE2 expression in the thoracic aorta of aged WKY rats. 

AT_2_R-mediated relaxation is a well-established effect in isolated resistance vessels [17–21]. Previous studies have shown AT_2_R-mediated vasodilatation in adult conscious rats [11–14, 22]. The AT_2_R-mediated reduction in blood pressure was likely to be a result of direct vasodilatation, rather than a result of decrease in cardiac output, as CGP42112 increased mesenteric and renal conductance in SHR, which was indicative of regional vasodilatation [13]. Furthermore, it is well documented that, in order to unmask any AT_2_R-mediated vasodilatation, there needs to be a removal of a tonic AT_1_R-mediated vasoconstriction induced by endogenous Ang II [23]. 

In the current study, acute Ang (1-7) infusion against a background of AT_1_R blockade resulted in a decrease in MAP in adult WKY male rats, and this Ang (1-7) response was mediated exclusively via AT_2_R in adult WKY male rats since the AT_2_R antagonist, PD123319, abrogated this Ang (1-7)-depressor response, which is consistent with previous findings obtained in both SHR and WKY rats [11]. Of note, the Ang (1-7) antagonist, A779, failed to inhibit vasodepressor responses induced by Ang (1-7) during AT_1_R blockade in adult rats, which confirmed our previous study that also found a 10-fold higher dose of A779 failed to block Ang (1-7) [11]. Thus, at least in this adult model, an exclusive role for Ang (1-7) as an endogenous ligand for the AT_2_R was demonstrated. 

In contrast, in the aged setting, the vasodepressor effect of Ang (1-7) was mediated by both AT_2_R and *Mas*R stimulation. Moreover, both candesartan doses (0.01 and 0.1 mg/kg) were effective in unmasking Ang (1-7)-mediated vasodepressor responses in aged rats. These results are consistent a 10-fold lower dose of candesartan being used to reveal Ang (1-7)-mediated vasodepressor effects via AT_2_R in SHR compared with WKY rats [11] and point towards an increased sensitivity to AT_1_R blockade in aged rats, as we have noted previously [24]. Increased vascular expression of AT_2_R in aging was seen in mesenteric resistance arteries [25] and in thoracic aorta [16]. Thus, Ang (1-7) infusion reduced MAP via AT_2_R in aged WKY rats irrespective of the background dose of candesartan. However, there are numerous reports suggesting the *Mas*R as the functional binding site for Ang (1-7) [7]. For example, Peiro et al. (2007), observed comparable impairment in Ang (1-7)-mediated vasorelaxation as a result of pharmacologic or genetic inhibition of *Mas*R using A779 and *Mas*R-deficient mice, respectively [26]. However, Ang (1-7) evoked vasorelaxation in pig coronary arteries that was attenuated by the AT_2_R antagonist, PD123319, suggesting an AT_2_R involvement [27]. Subsequent studies confirmed that Ang (1-7) can mediate its effects via AT_2_R [8–10]. Ang (1-7)-stimulated NO release in bovine aortic endothelial cells was markedly attenuated by AT_2_R inhibition (*∼*90%) [28, 29] and to a lesser extent by *Mas*R inhibition (*∼*50%) [28], suggesting activation of multiple receptors by Ang (1-7) which is also consistent with Ang (1-7)-stimulated arachidonic acid release in rabbit vascular smooth muscle cells [30]. 

**Figure 6:**
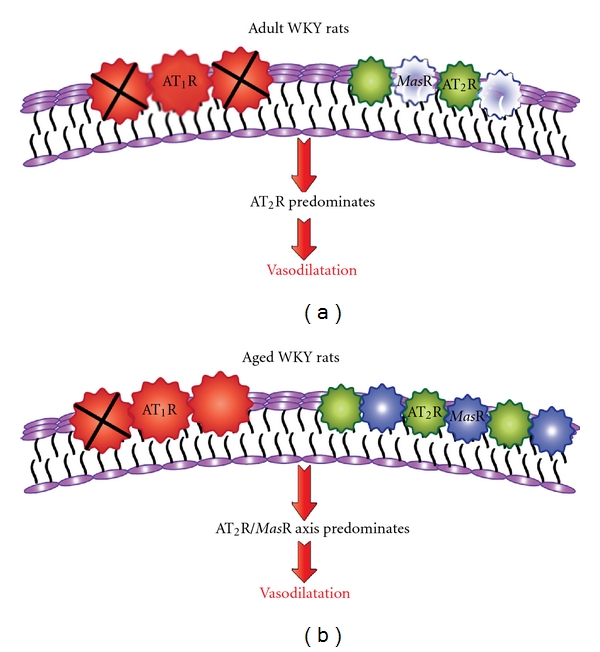
Schema depicting differential mechanisms of Ang (1-7)-mediated vasodepressor effect in adult and aged candesartan-treated rats. AT_1_R expression was similar in aortae from adult and aged rats whereas there was upregulation of AT_2_R, *Mas*R, and ACE2. Therefore, a lower level of AT_1_R blockade with candesartan (**X**) was required in aged animals (b) compared with adult animals (a) in order to unmask the vasodilator axis. *Mas*R was not functionally active in adult rats.

More recently, we have demonstrated that chronic treatment with Ang(1-7) was both vaso- and atheroprotective in Apolipoprotein E-deficient mice via both *Mas*R and AT_2_R [31]. Similarly, in the current study, we found that Ang (1-7) evoked a vasodepressor responses in aged candesartan-treated rats that was sensitive to both the AT_2_R antagonist PD123319 and the *Mas*R antagonist A779. This finding suggests that, unlike that in adult normotensive rats, Ang (1-7) can act via AT_2_R and/or *Mas*R during aging. Therefore, we also examined relative expression levels of the AT_1_R, AT_2_R, and *Mas*R as well as ACE2 to determine if this could account for the age-related differences in the cardiovascular effects of Ang (1-7). We have now confirmed an increased AT_2_R expression in aortae from aged WKY rats [16], and in addition we have shown, for the first time, a marked increase in expression levels of both *Mas*R and ACE2 in aortic sections from aged WKY rats. Future studies will need to confirm these findings using RT-PCR. These changes in ATR subtype expression fit with our *in vivo *results and also with other evidence for increased AT_2_R function in aging. For example, PD123319 can potentiate AT_1_R-mediated contractions, which is an indirect measure of AT_2_R relaxation [32, 33], and this “PD123319 potentiation” was enhanced in human coronary microvessels and was positively correlated with age [34]. To our knowledge, there are no reported functional correlates for enhanced Ang (1-7) in aging. At the same time, there was no difference in the expression levels of AT_1_R between adult and aged WKY rats, although a lower level of the AT_1_R block was required to unmask the depressor effect of Ang (1-7) in aged rats. One possible explanation for this difference between aged and adult WKY rats is due to the presence of several potential vasodilator pathways (AT_2_R, *Mas*R) resulting in preserved vasodilatation in aged WKY rats. This hypothesis is strengthened by the increased ratio of non-AT_1_R components to AT_1_R in aged WKY rats (Figure 6). 

In conclusion we have found that Ang (1-7)-mediated vasodepressor activity is preserved with aging. Thus, we can postulate that an increased AT_2_R/*Mas*R/ACE2 vasodilator axis relative to AT_1_R in aged rats is in part responsible for the ability of Ang (1-7) to operate via multiple mechanisms in aging, as opposed to only AT_2_R in adult normotensive candesartan-treated rats.

## Figures and Tables

**Table 1:** Resting MAP recorded on separate days before drug treatments, as indicated.

Treatment	MAP (mmHg)
Group 1 (*n* = 4)	
Saline	132 ± 14
Ang-(1-7) (15 pmol/kg/min)	124 ± 4
Candesartan (0.01 mg/kg)	131 ± 6
Ang-(1-7) and candesartan	134 ± 4

Group 2 (*n* = 7)	
Saline	131 ± 13
Ang-(1-7) (15 pmol/kg/min)	136 ± 13
Candesartan (0.01 mg/kg)	126 ± 15
Ang-(1-7) and candesartan	136 ± 16

Group 3 (*n* = 6)	
Ang-(1-7) (15 pmol/kg/min)	138 ± 8
Candesartan (0.1 mg/kg)	135 ± 8
Ang-(1-7) and candesartan	139 ± 11
Ang-(1-7), candesartan, and PD123319 (50 *μ*g/kg/min)	139 ± 5

Group 4 (*n* = 7)	
Ang-(1-7) (15 pmol/kg/min)	143 ± 10
Candesartan (0.1 mg/kg)	142 ± 10
Ang-(1-7) and candesartan	142 ± 9
Ang-(1-7), candesartan, and PD123319 (50 *μ*g/kg/min)	137 ± 9

Group 5 (*n* = 8)	
Ang-(1-7) (15 pmol/kg/min)	128 ± 11
Candesartan (0.1 mg/kg)	122 ± 10
Ang-(1-7) and candesartan	133 ± 5
Ang-(1-7), candesartan, and A779 (15 pmol/kg/min)	132 ± 2

Group 6 (*n* = 8)	
Ang-(1-7) (15 pmol/kg/min)	129 ± 5
Candesartan (0.01 mg/kg)	126 ± 10
Ang-(1-7) and candesartan	132 ± 6
Ang-(1-7), candesartan, and A779 (15 pmol/kg/min)	125 ± 8

Values are ± SEM.
